# Prediction of prognosis and pathologic grade in follicular lymphoma using ^18^F-FDG PET/CT

**DOI:** 10.3389/fonc.2022.943151

**Published:** 2022-07-28

**Authors:** Hongyan Li, Min Wang, Yajing Zhang, Fan Hu, Kun Wang, Chenyang Wang, Zairong Gao

**Affiliations:** ^1^ Department of Nuclear Medicine, Union Hospital, Tongji Medical College, Huazhong University of Science and Technology, Wuhan, China; ^2^ Hubei Province Key Laboratory of Molecular Imaging, Wuhan, China

**Keywords:** follicular lymphoma, ^18^F-FDG PET/CT, prognosis, pathologic grade, TLG

## Abstract

**Purpose:**

We investigated the utility of a new baseline PET parameter expressing lesion dissemination and metabolic parameters for predicting progression-free survival (PFS) and pathologic grade in follicular lymphoma (FL).

**Methods:**

The baseline ^18^F-FDG PET/CT images of 126 patients with grade 1–3A FL were retrospectively analyzed. A novel PET/CT parameter characterizing lesion dissemination, the distance between two lesions that were furthest apart (*D*_max_), was calculated. The total metabolic tumor volume and total lesion glycolysis (TLG) were computed by using 41% of the maximum standardized uptake value (SUV_max_) thresholding method.

**Results:**

The 5-year PFS rate was 51.9% for all patients. In the multivariate analysis, high *D*_max_ [*P* = 0.046; hazard ratio (HR) = 2.877], high TLG (*P* = 0.004; HR = 3.612), and elevated serum lactate dehydrogenase (*P* = 0.041; HR = 2.287) were independent predictors of PFS. A scoring system for prognostic stratification was established based on these three adverse factors, and the patients were classified into three risk categories: low risk (zero to one factor, *n* = 75), intermediate risk (two adverse factors, *n* = 29), and high risk (three adverse factors, *n* = 22). Patients in the high-risk group had a shorter 3-year PFS (21.7%) than those in the low- and intermediate-risk groups (90.6 and 44.6%, respectively) (*P* < 0.001). The C-index of our scoring system for PFS (0.785) was superior to the predictive capability of the Follicular Lymphoma International Prognostic Index (FLIPI), FLIPI2, and PRIMA-Prognostic Index (C-index: 0.628–0.701). The receiver operating characteristic curves and decision curve analysis demonstrated that the scoring system had better differentiation and clinical utility than these existing indices. In addition, the median SUV_max_ was significantly higher in grade 3A (36 cases) than in grades 1 and 2 FL (90 cases) (median: 13.63 vs. 11.45, *P* = 0.013), but a substantial overlap existed (range: 2.25–39.62 vs. 3.17–39.80).

**Conclusion:**

TLG and *D*_max_ represent two complementary aspects of the disease, capturing the tumor burden and lesion dissemination. TLG and *D*_max_ are promising metrics for identifying patients at a high risk of progression or relapse. Additionally, SUV_max_ seems to have some value for distinguishing grade 3A from low-grade FL but cannot substitute for biopsy.

## Introduction

Follicular lymphoma (FL) is one of most frequent subtypes of non-Hodgkin lymphoma (NHL) in the United States and Western Europe, accounting for around 22% of all NHLs. In China, the incidence of FL is lower than in Western countries, constituting 2.5–6.6% of all NHL cases (1, 2). The World Health Organization (WHO) classifies FL into grades 1 and 2, 3A, and 3B (3). Grades 1 and 2 are considered as indolent (slow-growing), whereas grade 3B has an aggressive course and is managed as diffuse large B-cell lymphoma (DLBCL) (4, 5). However, the optimal treatment for grade 3A FL is controversial (6, 7). Grade 3A FL has been suggested to be on the same continuum as grades 1 and 2 (8), and the contrasting findings revealed that gene expression profiling demonstrates a close relationship between FL 3A and 3B, but distinct from grades 1 and 2 (9). Prognostic models based on histologic grade and other factors have indicated that grade 3A is associated with a poor prognosis (10, 11). However, histologic grade (grades 1 and 2 *versus* grade 3A) arguably does not predict the disease outcome (12, 13). Nonetheless, FL grade plays an important role in treatment choice. An accurate assessment of histologic grade is challenging because of the heterogeneity of the disease and high inter-reader variability (14).

Although the survival of FL patients has markedly improved since the introduction of rituximab combined with chemotherapy, 20% of patients experience disease recurrence within 2 years, with a 5-year overall survival (OS) of just 50% (15, 16). The most common prognostic indices in current use, including the Follicular Lymphoma International Prognostic Index (FLIPI) (17) and FLIPI2 (18), cannot accurately identify patients who are at a high risk of progression or relapse (11, 19). PRIMA-Prognostic Index (PRIMA-PI) (20), a simplified scoring system including β2-MG (β2-microglobulin) and bone marrow involvement, has recently been proposed for patients treated with immunochemotherapy, but its development time is short, and the accuracy of bone marrow biopsy is still insufficient (21), so its application value still needs further validation.

^18^F‐fluorodeoxyglucose positron emission tomography/computed tomography (^18^F-FDG PET/CT) is recommended for FL staging and treatment response monitoring (22, 23). Total metabolic tumor volume (TMTV) and total lesion glycolysis (TLG) are new PET/CT metabolic parameters reflecting whole-body tumor burden that are becoming increasingly important for the prognostic assessment of lymphomas (24, 25). High baseline TMTV or TLG is associated with a significantly shorter progression-free survival (PFS) or OS in FL patients and has improved risk stratification (26–29). However, these metabolic parameters do not provide information on the spatial distribution of lesions throughout the body. The distance between two lesions that are the furthest apart (*D*_max_) has been recommended as a novel PET metric for describing tumor dissemination in DLBCL patients. A high *D*_max_ has been linked to an unfavorable prognosis and was shown to complement the prognostic performance of TMTV in advanced-stage DLBCL (30). We conjecture that the prognostic value of the metabolic parameters in FL might be improved by combining with the feature characterizing lesion dissemination.

Given the challenges of histological grading of FL, Major et al. (31) have focused on whether PET/CT can be used as an adjunct to biopsy grading of FL. The results showed that the maximum standardized uptake (SUV_max_), TMTV, and TLG were capable of differentiating grade 3A from low-grade (grade 1/2) FL, although this was based on a small number of cases, especially in the grade 3A group (11 patients).

Hence, the purpose of this study was to explore whether the new metric reflecting tumor dissemination and metabolic parameters derived from the baseline ^18^F-FDG PET/CT can be used to predict prognosis and histologic grade in patients with grades 1–3A FL.

## Materials and methods

### Patients

This study was approved by the institutional ethics board, and written informed consent was waived because of the retrospective nature.

We carried out a retrospective review of 126 FL patients undergoing ^18^F-FDG PET/CT scanning before treatment between February 2013 and December 2020. The inclusion criteria were as follows: (1) age >18 years and (2) histologic diagnosis of grades 1–3A FL according to the WHO classification (3). The exclusion criteria were as follows: (1) patients with other malignant tumors, (2) elevated fasting blood glucose level (≥200 mg/dl), and (3) histologically confirmed grade IIIB FL or concurrent DLBCL.

### PET/CT scanning

PET/CT scanning was performed using the Discovery VCT system (GE Healthcare, Milwaukee, WI, USA). Whole-body PET/CT scans (from the skull base to the upper thighs) were performed approximately 60 min after an intravenous injection of 3.7–4.4 MBq/kg ^18^F-FDG. CT data were used for attenuation correction, and corrected PET images were reconstructed using an ordered-subset expectation maximization iterative reconstruction algorithm. The CT and PET images were merged.

### Image analysis

PET/CT image data in DICOM format were used for functional parameter measurements using the AW workstation (AW4.6; GE Healthcare). The images were analyzed by two experienced nuclear medicine physicians. The highest ^18^F-FDG uptake in lesions was regarded as the SUV_max_ of the patient. MTV was delineated using the 41% SUV_max_ threshold method as recommended by the European Association of Nuclear Medicine (32). TMTV was defined as the sum of MTVs of all lesions. TLG was calculated as the sum of the product of MTV and SUV_mean_ of every individual lesion. Bone marrow involvement was considered in volume measurement only if there was focal uptake. Spleen was considered as involved if there was focal uptake or diffuse uptake higher than 150% of the liver background (26, 33). The site of the lesion’s SUV_max_ was regarded as the lesion’s position. *D*_max_ was calculated as the distance between two lesions that were furthest apart (30, 34). If the patient had only one lesion, the *D*_max_ value was denoted as 0 cm.

### Statistical analysis

The R software (version 3.6.2, https://www.r-project.org) was used for statistical analysis. A *P*-value <0.05 was considered statistically significant. All continuous variables are reported as mean ± SD or median when appropriate, and categorical variables are expressed as numbers and percentages. Differences in continuous variables were evaluated with the independent *t*-test or Mann–Whitney *U*-test, and categorical data were compared with the *χ* ^2^ test or Fisher’s exact test.

PFS was calculated as the time interval from initial diagnosis until disease relapse, progression, death, or the last follow-up. The X-tile software (version 3.6.1, Yale University, New Haven, CT, USA) was used to identify the optimal cutoff values for PET/CT parameters (35). The survival curves for PFS were plotted using the Kaplan–Meier method. Variables with *P <*0.05 in the univariate Cox analysis were included in the multivariate Cox regression model. A novel scoring system was established based on the number of risk factors for PFS. Harrell’s concordance index (C-index) was used to evaluate the performance of the model. A calibration plot comparing the relationship between the predicted and observed probabilities was used to assess the performance of the predictive model. The C-index, survival receiver operating characteristic (ROC) curves, and decision curve analysis (DCA) were used to compare the model’s predictive and discriminatory performance with that of FLIPI, FLIPI2, and PRIMA-PI.

To determine the optimal cutoff values of SUV_max_ and the platelet count for predicting the pathologic grade, ROC curves were plotted using MedCalc (Version 20.009). Multivariate logistic regression analysis was performed to identify factors that could predict the pathologic grade.

## Results

### Patient characteristics

The baseline clinical characteristics of the 126 patients (median age: 53 years, range: 21–76 years) are shown in **Table 1**. There were 90 patients with low grade and 36 with grade 3A disease. The patients were treated with rituximab plus cyclophosphamide, doxorubicin, vincristine, and prednisone (R-CHOP; *n* = 76); rituximab plus bendamustine (*n* = 14); CHOP (*n* = 9); rituximab plus cyclophosphamide, doxorubicin liposome, vincristine, and prednisone (*n* = 5); rituximab plus cyclophosphamide, vincristine, and prednisone (*n* = 4); rituximab plus fludarabine (*n* = 3); rituximab plus lenalidomide (*n* = 2); rituximab alone (*n* = 2); radiotherapy only (*n* = 2); and by the watch-and-wait approach (*n* = 9). After a median follow-up of 41 months (range: 1–102 months), 27 patients had a progressive disease but were alive, 15 patients died of this disease, and 84 patients were in complete remission or had a stable disease. The 5-year PFS rate for all patients was 51.9%.

**Table 1:** Characteristics of follicular lymphoma patients.

Characteristic	Number %
Age (years)	
<60	88 69.8
≥60	38 30.2
Gender	
Female	63 50.0
Male	63 50.0
Height (mean ± SD)	165.4 ± 8.2
B symptoms	
Absence	111 88.1
Presence	15 11.9
Histologic grade	
1 to 2	90 71.4
3A	36 28.6
Bone marrow involvement	
Absence	65 51.6
Presence	61 48.4
Ann Arbor stage	
I to II	22 17.5
III to IV	104 82.5
Number of nodal sites	
0–4	46 36.5
>4	80 63.5
LodLIN (cm)	
≤6	92 73.0
>6	34 27.0
Hemoglobin level (g/dl)	
≥12	81 64.3
<12	45 35.7
Platelet count	
≥150 × 10^9^/L	92 73.0
<150 × 10^9^/L	34 27.0
Serum LDH	
Normal	99 78.6
Elevated	27 21.4
β2-microglobulin	
Normal	85 67.5
Elevated	41 32.5
FLIPI	
Low risk	27 21.4
Intermediate risk	48 38.1
High risk	51 40.5
FLIP2	
Low risk	26 20.6
Intermediate risk	65 51.6
High risk	35 27.8
PRIMA-PI	
Low risk	52 41.3
Intermediate risk	33 26.2
High risk	41 32.5

LodLIN, longest diameter of the largest node; LDH, lactate dehydrogenase; FLIPI, Follicular Lymphoma International Prognostic Index; PRIMA-PI, PRIMA-Prognostic Index.

### Prognostic factors of PFS

**Figure 1:**
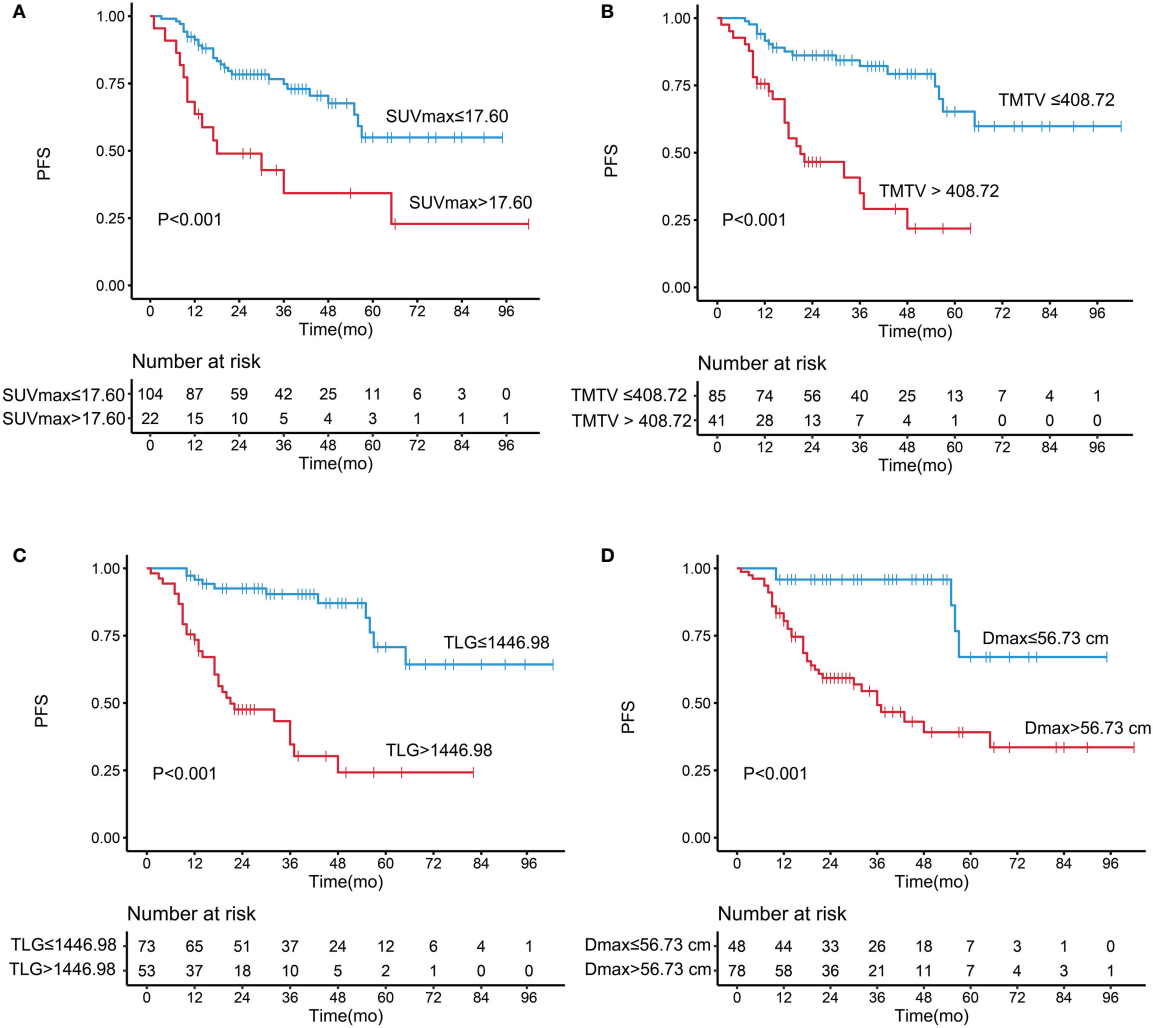
Kaplan–Meier analysis of progression-free survival according to **(A)** maximum standardized uptake value, **(B)** total metabolic tumor volume, **(C)** total lesion glycolysis, and **(D)** *D*_max._.

The optimal cutoff values of SUV_max_, TMTV, TLG, and *D*_max_ for PFS were 17.60, 408.72 cm^3^, 1446.98, and 56.73 cm, respectively. The univariate analysis showed that increased LDH level, increased β2-MG level, Hb <12 g/dl, SUV_max_ >17.60, TMTV >408.72 cm^3^, TLG >1,446.98, and *D*_max_ *>*56.73* cm* were associated with a significantly shorter PFS. The Kaplan–Meier curves and the univariate analysis results are presented in **Figure 1** and **Table 2**.

**Table 2:** Variables associated with progression-free survival (PFS) in the univariate analysis.

	PFS		
Variables	HR (95% CI)		*P*-value
Age (≥60 vs. <60)	1.158 (0.614–2.185)		0.651
Gender (male vs. female)	1.428 (0.776–2.626)		0.252
B symptoms (presence vs. *a*bsence)	0.891 (0.347–2.289)		0.810
Histologic grade (3A vs. 1 to 2)	1.375 (0.724–2.613)		0.331
Bone marrow involvement (presence vs. absence)	1.430 (0.778–2.629)		0.249
Ann Arbor stage (III to IV vs. I to II)	2.124 (0.831–5.428)		0.116
LDH (normal vs. elevated)	4.861 (2.495–9.472)		<0.001
β2-MG (normal vs. elevated)	4.009 (2.167–7.418)		<0.001
Hb (g/dl) (<12 vs. ≥12)	1.863 (1.017–3.416)		0.044
Platelet count (<150 × 10^9^/L vs. ≥150 × 10^9^/L)	1.747 (0.927–3.291)		0.081
Number of nodal sites (>4 vs. 0–4)	1.583 (0.820–3.058)		0.171
LodLIN (cm) (>6 vs. ≤6)	1.702 (0.880–3.290)		0.114
SUV_max_ (>17.60 vs. ≤17.60)	2.983 (1.566–5.681)		<0.001
TMTV (>408.72 cm^3^ vs. ≤408.72 cm^3^)	4.622 (2.450–8.717)		<0.001
TLG (>1,446.98 vs. ≤1,446.98)	6.736 (3.324–13.650)		<0.001
*D*_max_ (>56.73 cm vs. ≤56.73 cm)	6.344 (2.484–16.200)		<0.001
FLIPI			0.011
Low risk	Reference		
Intermediate risk	1.330 (0.498–3.552)		0.570
High risk	2.966 (1.207–7.289)		0.018
FLIP2			0.052
Low risk	Reference		
Intermediate risk	1.934 (0.729–5.136)		0.185
High risk	3.182 (1.164–8.702)		0.024
PRIMA-PI			<0.001
Low risk	Reference		
Intermediate risk	1.416 (0.568–3.530)		0.455
High risk	3.939 (1.911–8.120)		<0.001

LodLIN, longest diameter of the largest node; Hb, hemoglobin; LDH, lactate dehydrogenase; β2-MG, β2-microglobulin; SUV, standardized uptake value; TMTV, total metabolic tumor volume; TLG, total lesion glycolysis; D_max_, the largest distance between two lesions; FLIPI, Follicular Lymphoma International Prognostic Index; PRIMA-PI, PRIMA-Prognostic Index.

A high *D*_max_ was associated with a significantly shorter PFS [hazard ratio (HR) = 6.344, 95% confidence interval (95% CI) = 2.484–16.200, *P* < 0.001]; the 5-year PFS was 67.1% in the low-*D*_max_ group and 39.1% in the high-*D*_max_ group. The univariate analysis showed that Ann Arbor stage had no significant effect on PFS (*P* = 0.116). In a subgroup analysis of patients with stage III/IV disease, *D*_max_ remained a prognostic factor for PFS (*P* < 0.001). The results indicate that *D*_max_ has a strong predictive power for PFS, which was better than that of Ann Arbor stage. In addition, there was no significant differences in height between the low- and high-*D*_max_ groups.

The C-index of TLG was 0.737, which was higher than TMTV (C-index = 0.681; *P* = 0.076) and SUV_max_ (C-index = 0.614; *P* = 0.006). These three metabolic parameters were entered into the multivariate Cox regression model, respectively (**Table 3**). The independent risk factors for PFS were *D*_max_ (HR = 3.511, *P* = 0.014), SUV_max_ (HR = 2.143, *P* = 0.030), and β2-MG (HR = 2.622, *P* = 0.017) in the SUV_max_ model; *D*_max_ (HR = 3.798, *P* = 0.009) and LDH (HR = 2.223, *P* = 0.045) in the TMTV model; and *D*_max_ (HR = 2.877, *P* = 0.046), TLG (HR = 3.612, *P* = 0.004), and LDH (HR = 2.287, *P* = 0.041) in the TLG model.

**Table 3:** Multivariate analysis of variables predictive of PFS.

	Including SUV_max_		Including TMTV		Including TLG	
Variables	HR (95% CI)	*P*-value	HR (95% CI)	*P*-value	HR (95% CI)	*P*-value
LDH	—	0.123	2.223 (1.019–4.847)	0.045	2.287 (1.036–5.052)	0.041
β2-MG	2.622 (1.186–5.798)	0.017	—	0.509	—	0.686
Hb	—	0.624	—	0.577	—	0.671
*D*_max_	3.511 (1.285–9.594)	0.014	3.798 (1.396–10.339)	0.009	2.877 (1.021–8.103)	0.046
SUV_max_	2.143 (1.064–4.317)	0.030				
TMTV			—	0.095		
TLG					3.612 (1.525–8.559)	0.004

HR, hazard ratio; CI, confidence interval; PFS, progression-free survival; SUV_max_, maximum standardized uptake value; TMTV, total metabolic tumor volume; TLG, total lesion glycolysis; Hb, Hemoglobin; LDH, lactate dehydrogenase; β2-MG, β2-microglobulin; D_max_, the largest distance between two lesions.

### Prognostic stratification for PFS

**Figure 2:**
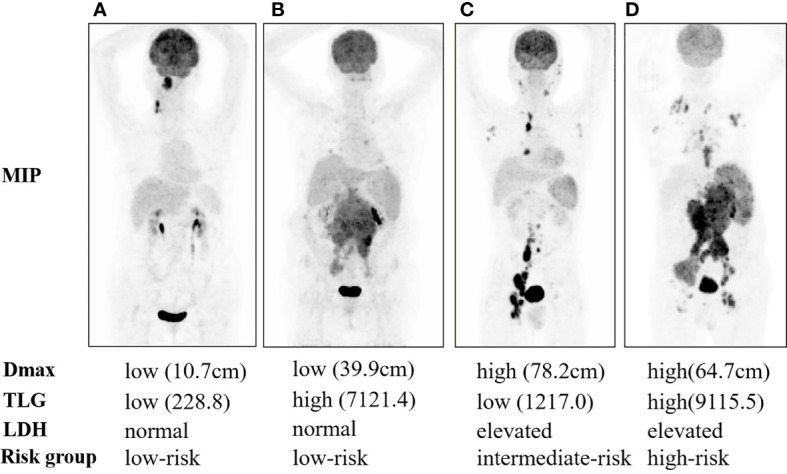
Maximal intensity projection of 1-3A follicular lymphoma patients with low risk **(A, B)**, intermediate risk **(C)**, and high risk **(D)**.

According to the results of the univariate and multivariate analyses, *D*_max_, TLG, and LDH were used to construct a scoring system for prognostic stratification. A novel scoring system was established based on the number of risk factors, and the patients were classified into three risk categories: low risk (zero to one adverse factor, *n* = 75), intermediate risk (two adverse factors, *n* = 29), and high risk (three adverse factors, *n* = 22). Examples of the three subgroups using maximal intensity projection on PET/CT images are shown in **Figure 2**.

**Figure 3:**
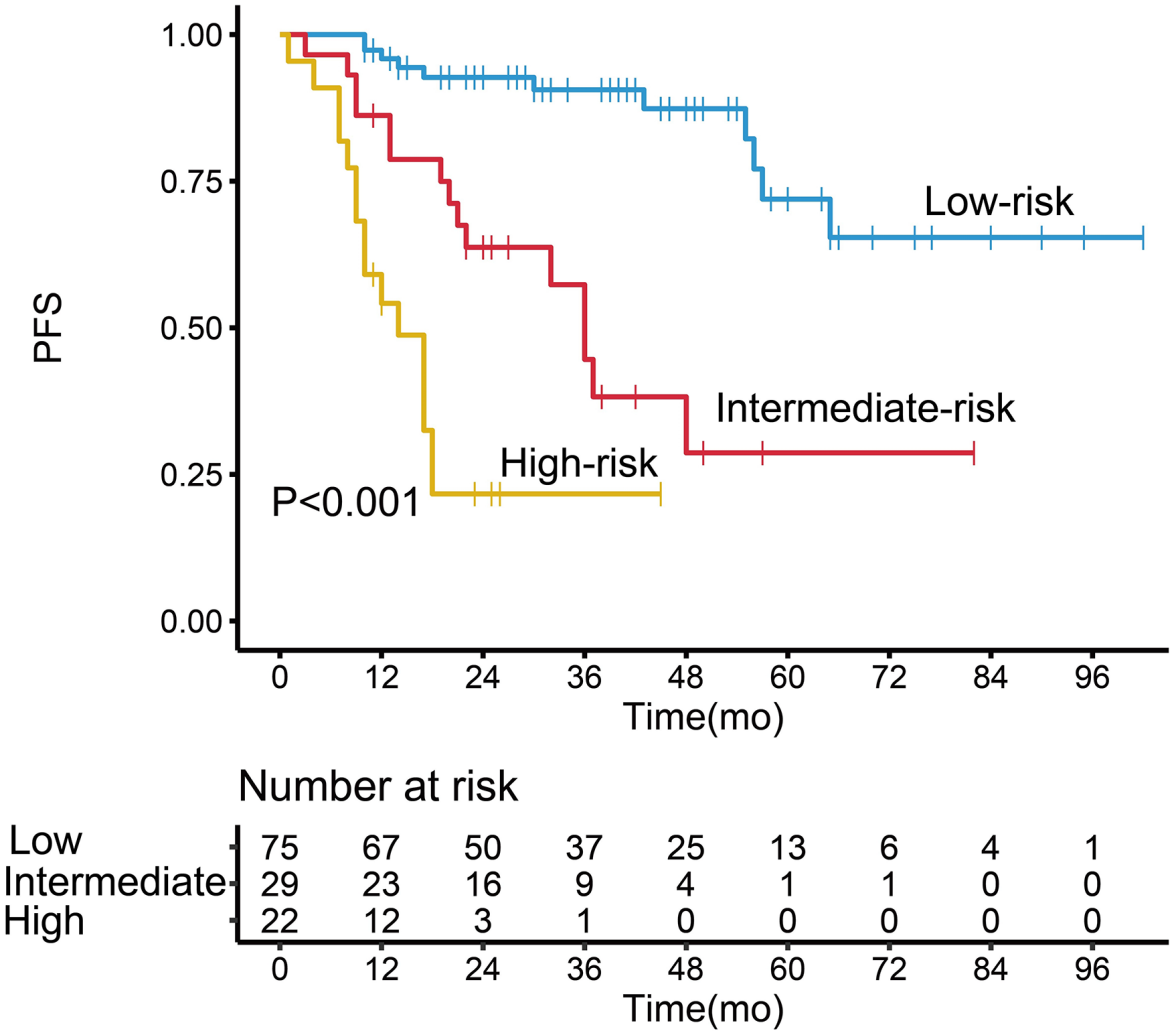
Kaplan–Meier survival analysis of progression-free survival in follicular lymphoma patients according to the potential grading system.

**Figure 4:**
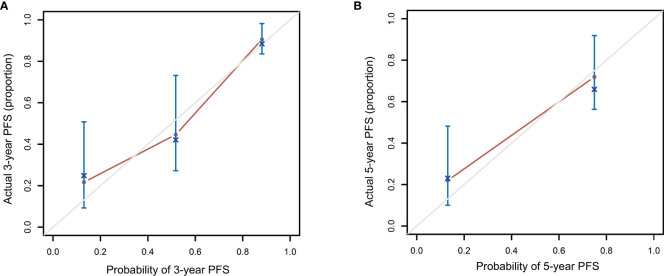
Calibration plot comparing the observed and predicted progression-free survival probabilities at 3 **(A)** and 5 **(B)** years.

Patients in the high-risk group had a shorter 3-year PFS (21.7%) than those in the low- and intermediate-risk groups (90.6 and 44.6%, respectively) (*P* < 0.001, **Figure 3**). The C-index for PFS of the scoring system was 0.785. The calibration plots for the 3- and 5-year PFS showed a good concordance between the predicted and the actual outcomes (**Figure 4**).

### Predictive accuracy for PFS of different prognostic scoring systems

**Figure 5:**
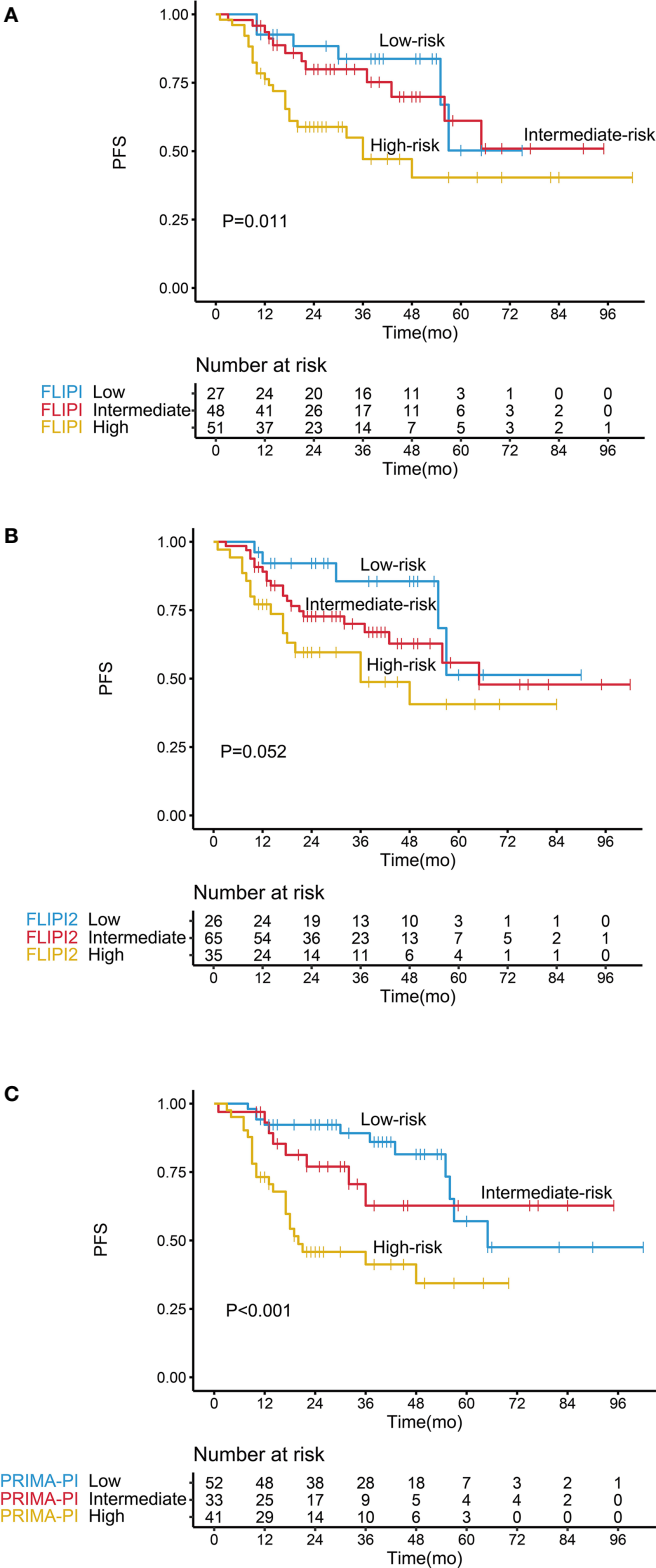
Kaplan–Meier survival analysis of progression-free survival in follicular lymphoma patients according to different prognostic models, including **(A)** FLIPI, **(B)** FLIPI2, and **(C)** PRIMA-PI.

FLIPI, FLIPI2, and PRIMA-PI showed a good performance in stratifying low- and high-risk patients according to PFS (**Figure 5** and **Table 2**). However, the three indices did not effectively discriminate between the intermediate- and low-risk patient groups (*P* > 0.05), and FLIPI2 did not show a good performance for discriminating between the intermediate- and high-risk groups (*P* > 0.05).

**Figure 6:**
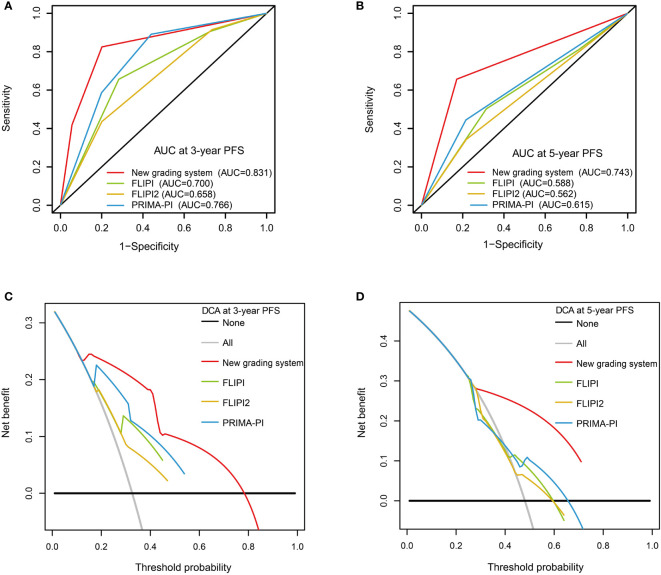
Receiver operating characteristic curves with the new grading system, FLIPI, FLIPI2, and PRIMA-PI for 3-year progression-free survival (PFS) **(A)** and 5-year PFS **(B)**. Decision curve analysis with the new grading system, FLIPI, FLIPI2, and PRIMA-PI for 3-year PFS **(C)** and 5-year PFS **(D)**.

The C-index of our scoring system for PFS was 0.785, which was significantly higher than that of FLIPI (0.650, *P* < 0.001), FLIPI2 (0.628, *P* < 0.001), and PRIMA-PI (0.701, *P* = 0.022) (**Table 4**). Similarly, the area under the curve (AUC) of our scoring system was higher than that of FLIPI, FLIPI2, and PRIMA-PI (**Figures 6A**, **B**). The DCA showed that our scoring system had better clinical utility than the three existing prognostic indices (**Figures 6C**, **D**).

**Table 4:** Comparative analysis of model performance for PFS between the new grading system and existing prognostic indexes.

Models	C-index (95% CI)	Compared with new grading system	
		Change (95% CI)	*P*-value
FLIPI	0.650 (0.570–0.730)	0.135 (0.078–0.198)	<0.001
FLIPI2	0.628 (0.547–0.709)	0.157 (0.081–0.234)	<0.001
PRIMA-PI	0.701 (0.622–0.780)	0.084 (0.011–0.155)	0.022
New grading system	0.785 (0.717–0.852)	—	—

PFS, progression-free survival; CI, confidence interval; FLIPI, Follicular Lymphoma International Prognostic Index; PRIMA-PI, PRIMA-Prognostic Index.

### New scoring system for patients receiving immunochemotherapy

In the whole cohort, 82.5% of patients (104/126) received immunochemotherapy. The multivariate Cox regression model for this population is summarized in **Supplementary Table S1**. These patients were divided into three risk subgroups according to our scoring system, and the Kaplan–Meier analysis showed that our model could discriminate among the three subgroups (**Supplementary Figure S1**). The C-index of the scoring system for PFS in the immunochemotherapy group was 0.774, which was higher than that of FLIPI (0.622), FLIPI2 (0.617), and PRIMA-PI (0.685).

### PET/CT parameters for predicting FL grade

The high-grade group showed a higher serum LDH level and a higher SUV_max_ than that in the low-grade group (*P* = 0.011, 0.013). Besides these, the low-grade group showed a higher platelet count than that in the high-grade group (*P* = 0.038) (**Table 5** and **Supplementary Figure S2**). In contrast, no significant differences were found in TMTV, TLG, *D*_max_, and other clinical indexes (all *P >*0.05).

**Table 5:** Comparison of clinical features and PET/CT parameters between grade 3A and low-grade follicular lymphoma.

	Grade 3A (*n* = 36)	Low grade (*n* = 90)	*P*-value
Age	52.25 ± 12.76	51.68 ± 11.24	0.804
Male (%)	20 (55.6)	43 (47.8)	0.430
Presence B symptoms (%)	4 (11.1)	11 (12.2)	0.862
Ann Arbor stage III to IV (%)	29 (80.6)	75 (83.3)	0.711
Hb level (g/dl) <12 (%)	17 (47.2)	28 (31.1)	0.088
Elevated LDH (%)	13 (36.1)	14 (15.6)	0.011
Elevated β2-MG (%)	13 (36.1)	28 (31.1)	0.588
Platelet count (10^9^/L) (median, interquartile range)	156 (143–199)	188 (151–241)	0.038
PET parameters (median, interquartile range)			
SUV_max_	13.63 (9.75–25.58)	11.45 (9.07–13.86)	0.013
TMTV	158.34 (27.81–417.24)	231.49 (104.86–576.96)	0.132
TLG	794.10 (217.92–3,220.61)	1,112.49 (476.48–2,583.25)	0.430
* D*_max_	56.76 (14.04–70.18)	66.99 (39.32–75.51)	0.190

Hb, hemoglobin; LDH, lactate dehydrogenase; β2-MG, β2-microglobulin; SUV_max_, maximum standardized uptake value; TMTV, total metabolic tumor volume; TLG, total lesion glycolysis; D_max_, the largest distance between two lesions.

Although the median SUV_max_ was higher in grade 3A FL than that in grades 1 and 2 (median: 13.63 vs. 11.45). There was an extensive overlap in SUV_max_ between the high- and low-grade groups (range: 2.25–39.62 vs. 3.17–39.80). The ROC curve analysis showed that an SUV_max_ of 17.38 was the optimal cutoff value (AUC: 0.642, sensitivity: 41.7%, specificity: 91.1%, PPV: 65.2%, and NPV: 79.6%; *P* = 0.020). The optimal cutoff value of platelet count was 154 × 10^9^/L (AUC: 0.619, sensitivity: 50.0%, specificity: 74.4%, PPV: 43.9%, and NPV: 78.8%; *P* = 0.035). The multivariate analysis identified SUV_max_ (*P* = 0.001) and platelet count (*P* = 0.017) as independent predictors of FL pathologic grade (**Supplementary Table S2**).

## Discussion

Identifying FL patients at a high risk of disease progression or recurrence and those with a high pathologic grade is essential for effective clinical management. Our study demonstrated that baseline ^18^F-FDG PET/CT values are useful for predicting PFS in FL. In the multivariate analysis, *D*_max_, TLG, and LDH were independent predictors of PFS. A novel scoring system for predicting PFS, which incorporated a new baseline PET parameter *D*_max_, reflecting lesion dissemination, along with the metabolic parameter TLG and serum LDH showed superior performance to FLIPI, FLIPI2, and PRIMA-PI. In addition, this study found that SUV_max_ was related to pathological grading, and PET/CT could be used as an auxiliary tool but cannot substitute for biopsy.

^18^F-FDG PET/CT provides important information about tumor burden. In the present study, baseline TLG was the most robust predictor of outcome in FL patients, whereas the predictive value of TMTV was limited. Consistent with our observations, a retrospective study found that TLG, rather than TMTV, was the independent prognostic factor for FL patient survival (27). A multicenter study reported that TMTV was a robust predictor of outcome in FL, and combining TMTV with FLIPI2 score showed a good performance in identifying patients at a high risk of early progression; however, these investigators did not explore TLG (26). Another study found that both TMTV and TLG were strong predictors of PFS and OS in FL (28). The discrepancy between these reports may be explained by the different cutoff values and thresholding methods used for TMTV or TLG, differences in the distribution of risk groups, and heterogeneity of the treatment strategies.

In this study, we used a new feature of PET/CT images, *D*_max_, to represent disease dissemination. *D*_max_ is a three-dimensional feature, which is easily measured and not influenced by the patient’s height. Unlike radiomic features, which are difficult to interpret from a biological perspective, *D*_max_ is a measure of the extent of the disease. *D*_max_ had a strong predictive power for PFS, which was better than that of the Ann Arbor stage, even among patients with advanced-stage FL. In line with our results, a high *D*_max_ predicted a shorter PFS and OS in DLBCL patients (30, 34). It has also been reported that a high *D*_max_ is a poor prognostic factor of HL (36). Unlike TMTV or TLG, which are derived from lesion contours, *D*_max_ is calculated as the distance between two lesions that were furthest apart. Most importantly, the measurements of *D*_max_ appeared to have good reproducibility and is thus broadly applicable.

Given the limitations of FLIPI and FLIPI2, other prognostic models for survival have been developed based on genomic or imaging data (19, 26, 29, 37, 38). In a multicenter study, TMTV and FLIPI2—which predicted markedly different PFS—were combined into a joint score (26). A prognostic model was developed for the same cohort that integrated baseline TMTV and end-of-induction PET (29). In the present study, we extracted two features from baseline PET/CT images—tumor burden and dissemination—representing two distinct and complementary aspects of the disease. We established a novel scoring system for predicting PFS based on *D*_max_, TLG, and LDH, which had a higher predictive accuracy than FLIPI, FLIPI2, and PRIMA-PI. Currently, most patients requiring treatment receive immunochemotherapy, which results in long-lasting remission and improved OS (5). Our prognostic scoring system was applicable and showed excellent performance in this population receiving immunochemotherapy. These results suggest that current prognostic indices can be further refined using tumor burden parameters and disease dissemination features obtained from PET/CT images. Due to the limited number of cases in this study, further research is needed to validate our results.

FDG uptake was shown to be higher in aggressive as compared to indolent NHL (39, 40). A widely varied SUV_max_ between low-grade and grade 3A FL was observed in many studies (31, 39). In a previous study, the cutoff value of SUV_max_ was 10.4, with 64% sensitivity and 74% specificity. In addition, TMTV and TLG had a comparable performance to SUV_max_ in making this distinction, with similar sensitivity and specificity values (31). However, TMTV and TLG could not discriminate low-grade from grade 3A FL in our study. The discrepancy between these findings and ours may be attributable to the differences in sample size and individual variability. The median SUV_max_ was significantly higher in the high-grade group than in the low-grade group (*P* = 0.013), but an extensive overlap existed, and there was a relatively low sensitivity for differentiating the grades of FL. Inevitably, some discordance in PET appearance may exist between the site of maximal FDG uptake and the actual site of tissue sampling. The histopathologic grading of different lesions in the same patients may be contrasting, resulting in an inconsistency between grading and clinical behavior. In addition, with the progression of the disease, some low-grade FL may be transformed into aggressive lymphoma (41, 42). The feasibility of using an absolute SUV_max_ cutoff value for grading FL is challenging. PET/CT may be a useful adjunct, but not a replacement for biopsy, to distinguish grade 3A from low-grade FL.

Platelet count was shown to be an independent prognostic indicator of outcome in peripheral T cell lymphoma and DLBCL (43, 44). However, platelet count was not a predictor of PFS in FL in the present study. The relationship between the platelet count and pathologic grade has been seldom discussed. We found that a low platelet count was an independent predictor of grade 3A FL. However, the underlying mechanism between the platelet count and the pathologic grade in FL needs further research.

Our study had some limitations. Firstly, this was a retrospective study with a relatively small sample size. Secondly, our predictive model was only for PFS. External validation in a larger population at multiple institutions is required.

## Conclusion

TLG and *D*_max_ obtained from PET/CT data represent two complementary aspects of the disease, capturing the whole-body tumor burden and lesion dissemination. TLG, *D*_max_, and serum LDH were independent prognostic factors of PFS. We established a novel scoring system for predicting PFS based on TLG, *D*_max_, and LDH, which showed a superior performance and clinical benefit compared to existing indices (FLIPI, FLIPI2, and PRIMA-PI).

Additionally, our results suggest that PET/CT may be a useful adjunct, but not a replacement, for biopsy in distinguishing grade 3A from low-grade FL.

## Data availability statement

The raw data supporting the conclusions of this article will be made available by the authors, without undue reservation.

## Ethics statement

The studies involving human participants were reviewed and approved by the Union Hospital, Tongji Medical College, Huazhong University of Science and Technology. Written informed consent for participation was not required for this study in accordance with the national legislation and the institutional requirements.

## Author contributions

HL: conceptualization, software, formal analysis, and writing—original draft preparation. MW: data curation, methodology, and writing—original draft preparation. YZ: visualization and investigation. FH: methodology and visualization. KW: software and validation. CW: data curation and validation. ZG: conceptualization, methodology, writing—review and editing, and supervision. All authors contributed to the article and approved the submitted version.

## Funding

This work was supported in part by the National Natural Science Foundation of China (No. 81771866).

## Conflict of interest

The authors declare that the research was conducted in the absence of any commercial or financial relationships that could be construed as a potential conflict of interest.

## Publisher’s note

All claims expressed in this article are solely those of the authors and do not necessarily represent those of their affiliated organizations, or those of the publisher, the editors and the reviewers. Any product that may be evaluated in this article, or claim that may be made by its manufacturer, is not guaranteed or endorsed by the publisher.
