# Supplementary material for: Prediction of prognosis and pathologic grade in follicular lymphoma using 18F-FDG PET/CT
Source: Front Oncol. 2022 Jul 28;12:943151. doi: 10.3389/fonc.2022.943151 (PMC9366037; doi:10.3389/fonc.2022.943151)
Supplement: Supplementary file 1 [file DataSheet_1.doc]

**Supplemental File**

**Supplemental Table 1.** Multivariate analysis of variables predictive of PFS for FL patients received immuno-chemotherapy

|  | **Including SUVmax** | | **Including TMTV** | | **Including TLG** | |
| --- | --- | --- | --- | --- | --- | --- |
| **Variables** | HR (95% CI) | P value | HR (95% CI) | P value | HR (95% CI) | P value |
| Hb | — | 0.433 | — | 0.600 | — | 0.633 |
| LDH | — | 0.099 | 2.564 (1.076-6.112) | 0.034 | 2.522 (1.053-6.036) | 0.038 |
| β2-MG | 2.381 (1.010-5.614) | 0.047 | — | 0.949 | — | 0.778 |
| Dmax | 2.844 (1.010-8.006) | 0.048 | — | 0.060 | — | 0.133 |
| SUVmax | — | 0.061 |  |  |  |  |
| TMTV |  |  | 2.983 (1.128-7.890) | 0.028 |  |  |
| TLG |  |  |  |  | 3.261 (1.276-8.332) | 0.014 |

Abbreviations: HR, hazard ratio; CI, confidence interval; PFS, progression-free survival; SUVmax, maximum standardized uptake value; TMTV, total metabolic tumor volume; TLG, total lesion glycolysis; Hb, Hemoglobin; LDH, lactate dehydrogenase; β2-MG, β2-microglobulin; Dmax, the largest distance between two lesions.

**Supplemental Table 2** multivariate logistic regression analyses for predicting follicular lymphoma grade

| Variables | Multivariate regression | | |
| --- | --- | --- | --- |
|  | OR | 95%CI | P value |
| LDH | — | — | 0.605 |
| Platelet count | 3.017 | 1.224-7.436 | 0.017 |
| SUVmax | 6.353 | 2.113-19.108 | 0.001 |

Abbreviations: LDH, lactate dehydrogenase; SUVmax, maximum standardized uptake value.


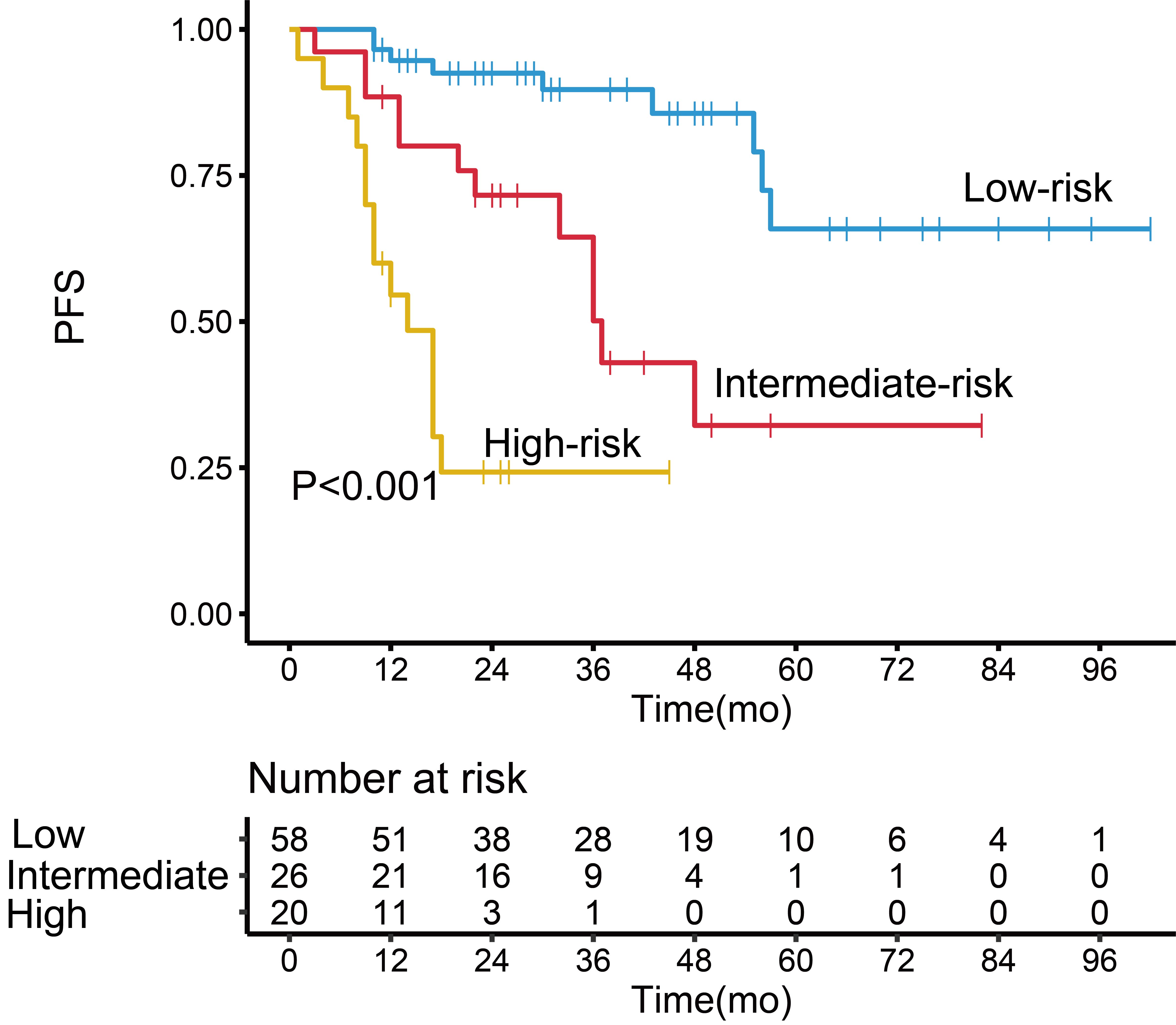


**Supplemental Fig. 1** Kaplan-Meier survival analysis of PFS in FL patients received immuno-chemotherapy according to the potential grading system.


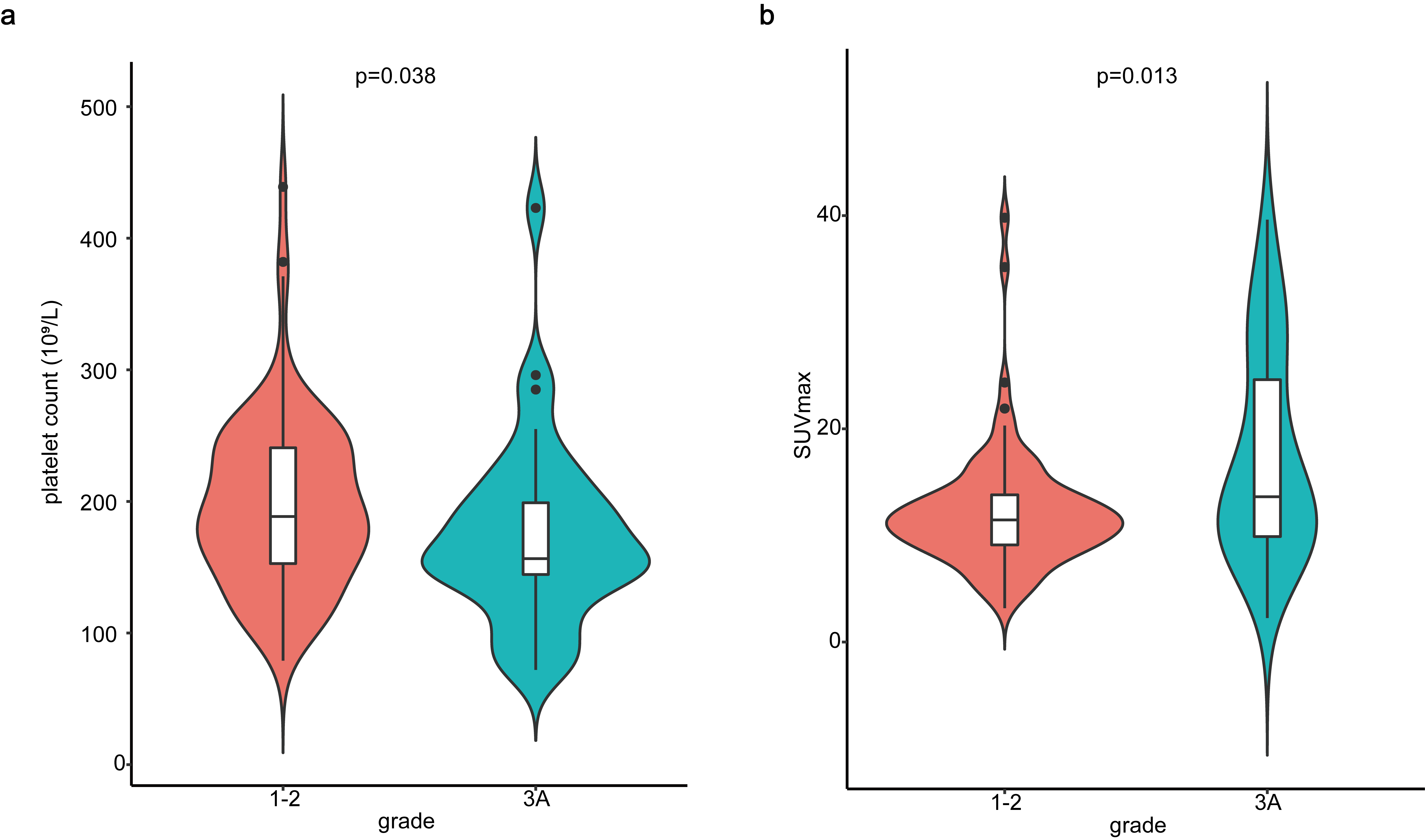


**Supplemental Fig. 2** Violin plot of platelet count (A) and SUVmax (B) in grade 1-2 and grade 3A FL group.
